# Supplementary material for: Tissue-specific targeting of DNA nanodevices in a multicellular living organism
Source: eLife. 2021 Jul 28;10:e67830. doi: 10.7554/eLife.67830 (PMC8360651; doi:10.7554/eLife.67830)
Supplement: Supplementary file 1. [file elife-67830-supp1.docx]

**Table 1. Sequences of oligonucleotides employed in this study.**

| **Name** | **Sequences 5'-3'** |
| --- | --- |
| R1^50^ | GGA CTC TGG GGT TCG AAA TGA CCG ACC AAG CGA CGC CCA ACC TGC CAT CC |
| R2^50^ | GGA TGG CAG GTT GGG CGT CGC TTG GTC GGT CAT TTC GAA CCC CAG AGT CC |
| R1^100^ | GGA CTC TGG GGT TCG AAA TGA CCG ACC AAG CGA CGC CCA ACC TGC CAT CAC GAG ATT TCG ATT CCA CCG CCG CCT TCT ATG AAA GGT TGG GCT TCG GAC C |
| R2^100^ | GGT CCG AAG CCC AAC CTT TCA TAG AAG GCG GCG GTG GAA TCG AAA TCT CGT GAT GGC AGG TTG GGC GTC GCT TGG TCG GTC ATT TCG AAC CCC AGA GTC C |
| D1^38^ | DBCO-ATCAACACTGCACACCAGACAGCAAGATCCTATATATA |
| D2^38^ | Alexa 647-TATATATAGGATCTTGCTGTCTGGTGTGCAGTGTTGAT |
| D1^50^ | TAT CAG TAG GTT CTT CAG AGT ATT GTC TCT TCC GTG TAT CAG TTA GCC TC-DBCO |
| D2^50^ | Alexa 647-GAG GCT AAC TGA TAC ACG GAA GAG ACA ATA CTC TGA AGA ACC TAC TGA TA |
| D1^100^ | TAT CAG TAG GTT CTT CAG AGT ATT GTC TCT TCC GTG TAT CAG TTA GCC TCG ACT CTC ATA GTG GAC GTA GAA CTC CAC ATC AGA TCA CTG GAA GAT CAT C-DBCO |
| D2^100^ | Alexa 647-GAT GAT CTT CCA GTG ATC TGA TGT GGA GTT CTA CGT CCA CTA TGA GAG TCG AGG CTA ACT GAT ACA CGG AAG AGA CAA TAC TCT GAA GAA CCT ACT GAT A |
| BIOTIN‐R‐CELL | Biotin‐AAA ATA CAT TTA TAT ATA TCC TAG TCC GAC CGC AGG ATC CTA TAA |
| R‐CELL | TAC ATT TAT ATA TAT CCT AGT CCG ACC GCA GGA TCC TAT AA |
| BIOTIN‐O3‐CELL | TTA TAG GAT CCT GCG GTC GGA CTA GGA TAT ATA TAA ATG TAA AAA‐Biotin |
| O3‐CELL | TTA TAG GAT CCT GCG GTC GGA CTA GGA TAT ATA TAA ATG TA |
| R1‐CELL | TTA TAG GAT CCT GCG GT |
| RM‐CELL | GCG GTC GGA CTA GGA TA |
| R2‐CELL | GGA TAT ATA TAA ATG TA |
| R3‐CELL | AGG ATC CTG CGG TCG GA |
| R4‐CELL | TCC TGC GGT CGG ACT AG |
| R5‐CELL | GCG GTC GGA CTA GGA TA |
| BIOTIN‐RR6‐CELL | Biotin‐AAA ATA CAT TTA TAT ATA TCC TAG TCC ATA AGA CCG CAG GAT CCT |
| R6‐CELL | AGG ATC CTG CGG TCT TAT GGA CTA GGA TAT ATA TAA ATG TA |
| BIOTIN‐R‐CELL‐33 | Biotin‐AAAATACATTTCCTAGTCCGACCGCAGGATCCTATAA |
| O3‐CELL‐33 | TTATAGGATCCTGCGGTCGGACTAGGAAATGTA |
| BIOTIN‐DS1‐CELL | Biotin‐AAA ATG CAG GGT ACG GTA CGG TAC GCC GGA CGC GAC TAG TTA CGG |
| DS2‐CELL | CCG TAA CTA GTC GCG TCC GGC GTA CCG TAC CGT ACC CTG CA |
| I4‐CELL | CCC CTA ACC CCT AAC CCC TAA CCC CAT ATA TAT CCT AGA ACG ACA GAC AAA CAG TGA TAA |
| I4‐COMP‐CELL | TTA TCA CTG TTT GTC TGT CGT TCT AGG ATA TAT ATT TTG TTA TGT GTT ATG TGT TAT |
| nD' | AT CAA CAC TGC ACA CCA GAC AGC TAG GAT CCT ATA A |
| nD^647^ | TTA TAG GA/iATTO647NN/ CCT AGC TGT CTG GTG TGC AGT GTT GAT |
| nD’^488^ | Alexa488-AT CAA CAC TGC ACA CCA GAC AGC TAG GAT CCT ATA A |
| nD’-9E | TCA CAC TGC ACA CTA CAG ACA GCA AGA TCCA |
| nD^647N^-9E | TGGA TCT TGC TGT CTG TAG TGT GCA GTG TGA |
| D_P_ | 5AmMC6/ATCAACACTGCACACCAGACAGCTAGGATCCTATAA |
| D_A_ | TTATAGGA/IATTO647NN/CCTAGCTGTCTGGTGTGCAGTGTTGAT |
